# Clinical Evaluation of a Custom Gene Panel as a Tool for Precision Male Infertility Diagnosis by Next-Generation Sequencing

**DOI:** 10.3390/life10100242

**Published:** 2020-10-15

**Authors:** Rossella Cannarella, Vincenza Precone, Giulia Guerri, Gian Maria Busetto, Gian Carlo Di Renzo, Sandro Gerli, Elena Manara, Astrit Dautaj, Matteo Bertelli, Aldo Eugenio Calogero

**Affiliations:** 1 Department of Clinical and Experimental Medicine, University of Catania, 95124 Catania, Italy; rossella.cannarella@phd.unict.it (R.C.); acaloger@unict.it (A.E.C.); 2 MAGI EUREGIO, 39100 Bolzano, Italy; vincenza_precone@yahoo.it (V.P.); elena.manara@assomagi.org (E.M.); matteo.bertelli@assomagi.org (M.B.); 3 MAGI’S LAB, 38068 Rovereto, Italy; giulia.guerri@assomagi.org; 4 Department of Urology, “Sapienza” University of Rome, Policlinico Umberto I, 00185 Rome, Italy; gianmaria.busetto@uniroma1.it; 5 Department of Surgical and Biomedical Sciences, University of Perugia, 06123 Perugia, Italy; giancarlo.direnzo@unipg.it (G.C.D.R.); sandro.gerli@unipg.it (S.G.); 6 EBTNA-LAB, Via delle Maioliche, 57/G, 38068 Rovereto, Italy

**Keywords:** male infertility, NGS diagnosis, defects of primary spermatogenesis, hypogonadotropic hypogonadism, primary ciliary dyskinesia

## Abstract

Background: Up to 15% of couples are infertile and male factor infertility accounts for approximately 50% of these cases. Male infertility is a multifactorial pathological condition. The genetic of male infertility is very complex and at least 2000 genes are involved in its etiology. Genetic testing by next-generation sequencing (NGS) technologies can be relevant for its diagnostic value in male infertile patients. Therefore, the aim of this study was to implement the diagnostic offer with the use of an NGS panel for the identification of genetic variants. Methods: We developed an NGS gene panel that we used in 22 male infertile patients. The panel consisted of 110 genes exploring the genetic causes of male infertility; namely spermatogenesis failure due to single-gene mutations, central hypogonadism, androgen insensitivity syndrome, congenital hypopituitarism, and primary ciliary dyskinesia. Results: NGS and a subsequent sequencing of the positive pathogenic or likely pathogenic variants, 5 patients (23%) were found to have a molecular defect. In particular, pathogenic variants were identified in *TEX11*, *CCDC39*, *CHD7*, and *NR5A1* genes. Moreover, 14 variants of unknown significance and 7 novel variants were found that require further functional studies and family segregation. Conclusion: This extended NGS-based diagnostic approach may represent a useful tool for the diagnosis of male infertility. The development of a custom-made gene panel by NGS seems capable of reducing the proportion of male idiopathic infertility.

## 1. Introduction

It is estimated that about 48.5 million couples worldwide are affected by infertility [1,2,3]. Epidemiological data have addressed to the male factor an etiologic role, alone or in combination, in the half of cases of couple’s infertility [4]. Genetic causes of male infertility, as well as the effects of their transmission to the offspring are still not fully understood [5]. The primary male infertility can be associated with abnormal semen or normal semen (idiopathic infertility). Instead, the secondary male infertility is due to systemic or syndromic genetic defects [6]. Disorders of sex development, ambiguous genitalia and androgen insensitivity, central hypogonadism, congenital hypopituitarism, and primary ciliary dyskinesia (PCD) can occur in syndromic forms [6,7,8,9,10,11]. Laboratory tests are of pivotal importance for a correct etiological classification of male infertility and for the choice of an effective treatment [12,13]. In addition to traditional first level diagnostic tests (seminal fluid analysis, hormone measurement, bacteriological analysis, and the search for anti-sperm antibodies), second level diagnostic tests such as sperm function tests and genetic analysis are useful for the assessment of male infertility [13]. In particular, genetic analysis is of relevance since about 15–30% of male infertility cases may recognize a genetic factor [14,15,16]. Genetic disorders related to male infertility include monogenic diseases and whole or partial chromosomal aberrations. Over 200 genetic conditions related to male infertility are reported in Online Mendelian Inheritance in Man (OMIM) database [17,18]. The identification of the genetic defect in the management of infertility allows also to establish the risk of its possible inheritance in preconception screening and the optimization of assisted fertilization techniques [19,20,21]. The genetic tests commonly used for male infertility diagnosis include karyotyping to detect numerical or structural abnormalities and PCR (polymerase chain reaction) to detect known genetic anomalies. However, it must be considered that the etiology remains elusive in about 40% of cases of male infertility (idiopathic forms) that, very likely, have a genetic unknown background [22,23]. Moreover, also it would be necessary to consider the role of recently discovered autosomal single-gene disorders in male infertility [24]. In fact, long been known chromosomal syndromes associated with infertility diagnosed by karyotype analysis, but only in recent years studies have been conducted on specific chromosomal regions and identified single genes linked to infertility problems. About 30% of infertility cases are still apparently *sine causa* and it is difficult for these to be syndromic conditions, but rather for conditions in which the reproductive defect is the only diagnostic indication [25,26]. Knowledge of the molecular defect attributable to single genes could lead to the development of targeted therapies to correct the genetic defect. The complexity of genetic tests for the diagnosis of infertility is continuously and rapidly growing thanks above all to the advent of new biomolecular and bioinformatics technologies. Within this scenario, the next-generation sequencing (NGS) technique can improve and personalize male infertility diagnosis. In addition, NGS can allow the simultaneous analysis of a large cohorts of infertile patients and the screening of multiple disease-causing variants of many genes [27]. The use of multigene panels can be a useful tool to improve the diagnostic framework of the patients [21,27]. Therefore, the aim of this study was to implement the diagnostic offer with the use of an NGS panel for the identification of genetic variants that may support a more accurate management of male infertility.

## 2. Results

Twenty-two male patients with infertility were analyzed with a NGS customized panel. Their median age was 37 years (range 19–56). The diagnostic suspects, made matching data coming from anamnesis, physical examination, hormone values, scrotal ultrasound, and/or sperm analysis, were primary spermatogenic defects (*n* = 16), central hypogonadism (*n* = 3), androgen insensitivity (*n* = 1), congenital hypopituitarism (*n* = 1), and primary ciliary dyskinesia (*n* = 1). For each patient, only the genes of the panel related to the diagnostic suspect were analyzed because the analysis was for diagnostic purposes. Genetic variants identified in the analyzed population by NGS are reported in Supplementary Table S1, whereas variants identified in the patients with a positive genetic diagnosis are reported in Table 1.

In clinical utility assessment, 5 patients (23%) resulted positive for the diagnostic test.

### 2.1. Case 1

Case 1 was a 41-year-old patient, trying to reach fatherhood in the last 2 years. The female partner was 32 years old and no female factor infertility was found. Patient’s physical examination was unremarkable, showing normal androgenization and no signs of genital malformation. Sperm analysis (which was performed according to the WHO 2010 criteria) showed oligoasthenozoospermia for three times. The mean (range) conventional sperm parameters were: 1.7 (0.36–4) million/mL for sperm concentration, 6.6 (2.5–14) million/ejaculate for total sperm count, 7 (3–10%) for sperm progressive motility, 35.3 (20–56) for total sperm motility, 5.7 (3–10%) for spermatozoa with normal form. Seminal fluid leukocytes were <1 million/mL in all specimens. Follicle-stimulating hormone (FSH) serum values were 8.1 IU/L. At scrotal ultrasound, testicular volumes were 14.7 mL and 14.1 mL for the right and the left testis, respectively. Male accessory gland infection, varicocele, or other acquired causes of male infertility were carefully assessed and could be excluded.

The patient was hence suspected of a defect of primary spermatogenic failure due to mutation in single genes. The analyzed sample showed the presence of two sequence alterations in the *TEX11* gene located in the chromosome X, namely the pathogenic variant p.(Val763Ala) (c.2288T > C) (rs200139216) [28] and the likely benign variant p.(Thr259Ile) (c.776C > T) (rs762957753). In addition, it has also been identified in *ZPBP* (Zona Pellucida Binding Protein) gene the likely benign variant p.(Arg17Pro) (c.50G > C) (rs202231065) in heterozygosity. *ZPBP* gene ex:1 (chr7:50093185-50093209) was re-sequenced by Sanger (NGS coverage: 4,5X).

### 2.2. Case 2

Case 2 was a 19-year-old male patient suspected of having a primary ciliary dyskinesia. Physical examination showed normal androgenization, no genital malformation, normal penile size, and testicular volume, but the presence of right varicocele. At instrumental examination, he had dextrocardia and bronchiectasia. The patient was healthy and did not have respiratory symptoms. Sperm analysis documented azoospermia. After scleroembolization, he had oligoasthenoteratozoospermia. Transmission electronic microscopy analysis documented abnormal connecting piece and the absence of outer dynein arms in the sperm axoneme. It the respiratory cilia it showed abnormal number and position of peripheral microtubules and of central pair. The analyzed sample showed the presence of three sequence alterations in *CCDC39* (Coiled-Coil Domain Containing 39) gene, namely the pathogenic variant c.610-2A > G (rs756235547) [29], a variant with uncertain pathogenic significance p.(Arg811Cys) (c.2431C > T) (rs574993914) and a variant probably benign p.(Thr182Ser) (c.545C > G) (rs112738198), all in heterozygosity. Moreover, it has also been identified in *CDC151* gene a variant with uncertain pathogenic significance p.(Thr205Ile) (c.614C > T) (rs35061520) in heterozygosity detected by Sanger sequencing. However, since this gene is associated with a recessive inheritance, it is unlikely that this mutation alone could cause PCD.

### 2.3. Case 3

Case 3 was a 26-year-old male patient with reversal central hypogonadism. At the age of 14 years old, due to the absence of puberty, he underwent to genetic testing and pituitary imaging, revealing abnormally low gonadotropins (the pituitary function was normal for the other pituitary hormones) and the absence of abnormalities at imaging. He was then prescribed gonadotropins. At the age of 22 years, the patient reached a complete degree of androgenization. Withdrawal from treatment revealed the persistence of low gonadotropins and the diagnosis of central hypogonadism was confirmed. No abnormality of smell sense was referred by the patient. He was then prescribed testosterone replacement therapy (TRT). At the age of 24 years, TRT was discontinued. At hormone testing, gonadotropins were found within the normal range. At scrotal ultrasound, testicular volumes were 5.8 mL and 5 mL, in the right and in the left testis, respectively. At the sperm analysis, a severe oligoasthenoteratozoospermia was found after 5 months from TRT withdrawal. Specifically, 5 spermatozoa in the whole specimen were found. None of them were motile and normally shaped. A severe left varicocele was found, and the patient was then counseled for scleroembolization.

At genetic testing, pathogenic variant p.(Thr917Met) (c.2750C > T) (rs1165711448) of the *CHD7* (Chromodomain-helicase-DNA-binding protein 7) gene was found [30]; it was confirmed by Sanger sequencing, and the variant with uncertain pathogenic significance p.(Ala369Val) c.1106C > T (rs771836971) in *FLRT3* (Fibronectin Leucine Rich Transmembrane Protein 3) gene, both in heterozygosity.

### 2.4. Case 4

Case 4 was a 36-year-old male patient searching for fatherhood in the last 3 years. The female partner had a child from a previous marital relationship and a female factor infertility was excluded. Physical examination was unremarkable, with no sign of hypo-androgenization or genital malformation. FSH serum levels were 4.1 IU/mL, luteinizing hormone (LH) 3.6 IU/mL, and total testosterone 5.7 ng/mL. At ultrasound, right and left testicular volumes were 14.1 mL and 14.9 mL, respectively. The patient underwent sperm analysis for 4 time that showed oligozoospermia and teratozoospermia (mean sperm concentration 7.6 million/mL, mean total sperm count 21.5 million/ejaculate, sperm with normal morphology 3%). Acquired causes of oligozoospermia were carefully excluded. The patient was then suspected of a defect of primary spermatogenic failure due to mutation in single genes. The pathogenic variant p.(Ala351Val) c.1052C > T (rs759071081) of the *NR5A1* (Nuclear Receptor Subfamily 5 Group A Member 1) gene [31] was found in heterozygosity by NGS.

### 2.5. Case 5

Case 5 was a 39-year-old patient counseled for primary male infertility. His wife and he had searched for childhood in the last two years. The patient’s wife was 35 years old and no female factor infertility was found. The physical examination of the patient was unremarkable, with normal androgenization and no sign of genital malformation. FSH serum levels were above the normal range (17.5 IU/mL) and, at ultrasound, the right and the left testicular volumes were 6.3 mL and 9.8 mL, respectively, indicating testicular hypotrophy. Two sperm analyses showed 11.5 million/mL for sperm concentration, 11.5 million/ejaculate for total sperm count, 7.5% for progressive motility, 50% for total motility, and 5.5% spermatozoa with normal morphology. These values indicated a diagnosis of oligoasthenozoospermia. After acquired causes were excluded, the patient underwent to genetic testing for the suspicious of primary spermatogenic failure due to mutation in single genes. The genetic test revealed the pathogenic variant in *NR5A1* gene p.(Val355Met) (c.1063G > A) (rs371701248) [31] in heterozygosity and other two not pathogenic variants: p.(Arg323Cys) c.967C > T (rs764712886) in *SEPT12* gene and p.(Ile2226Val) (c.6676A > G) (rs112505934) in *DNAH1* gene. Interestingly, one year later, the patient spontaneously made his wife pregnant.

The coverage performance by NGS for these subjects is shown in Table 2. Coding genomic regions that were sequenced with a coverage less than 10X were eventually re-sequenced using Sanger technology.

## 3. Discussion

Male infertility can be the result of non-genetic or genetic factors, and it is often multifactorial and/or polygenic [32]. Hundreds of genes implicated in the development and function of the male reproductive system and in spermatogenesis can be involved [33]. Additional genetic anomalies contributing to male infertility are constantly identified. The process of evaluating the genes involved in male infertility is essential to optimize genetic testing in reproductive medicine and to implement the therapeutic management based on the specific genomic features of the patient. Interestingly, clinicians can more accurately decide whether male infertile patients are candidates for surgical retrieval of spermatozoa for use in assisted fertilization techniques by the knowledge of the nature of the genetic variants [34]. Studies of identification of relevant mutations and polymorphisms by NGS screening technologies can be used for this purpose and easily incorporated into the clinical practice. Moreover, NGS technologies have made it possible to compact one or more tests into a single NGS-based analysis, thus reducing diagnostic costs and time [35].

We have successfully developed a genetic test based on NGS that covers the main male infertility causes [25,36,37,38]. We have analyzed a custom-made panel of 110 genes in 22 infertile patients (Table 3).

Five of them with suspicion of primary spermatogenesis failure, central hypogonadism, and primary ciliary dyskinesia resulted positive at the diagnostic test. Five variants with a clearly known pathogenic role were identified in *TEX11*, *CCDC39*, *CHD7*, and *NR5A1* genes. *TEX11* (Testis-expressed gene 11), mapping on the Xq13.1 chromosome, is a X-linked gene essential for meiotic recombination and chromosomal synapsis expressed only in male germ cells [39,40]. The role of *TEX11* in spermatogenesis has been recognized in knock-out mouse models and humans [28,41]. Currently, 47 *TEX11* variants are known, including 25 variants in azoospermic male and 22 variants in fertile subjects [28]. *CCDC39* (Coiled-Coil Domain-Containing 39), mapping on the chromosome 3q26.33, encodes for a protein involved in the motility of cilia and flagella. In particular, variations of *CCDC39* gene cause defects in the assembly of the nexin-dynein regulatory complex and inner dynein arms, and disruption of this periodicity. Axonemal disorganization and inner dynein arm defects represent about 12% of all primary ciliary dyskinesia cases [42]. *CHD7* is a gene, mapping on the 8q12.2 chromosome, associated with the CHARGE syndrome. It overlaps with Kallmann syndrome and central hypogonadism, since affects patients that show gonadotropin deficiency, showing that this gene is involved in puberty and reproduction [43]. Almost all *de novo* variants in *CHD7* occur in the paternal germline [44,45]. Finally, *NR5A1* (steroidogenic factor-1), a member of the nuclear receptor superfamily mapping on the chromosome 9q33.3, is a key transcriptional regulator of genes involved in the hypothalamic-pituitary-steroidogenic axis [46,47]. In mammalian testis determination and differentiation, *NR5A1* is a positive regulator of *SOX9* gene and anti-Müllerian hormone [48]. About 4% of patients with otherwise unexplained severe spermatogenic failure carry variants in *NR5A1* [49]. Variants of this gene are also a genetic cause of severer forms of male factor infertility, especially when associated with a history of cryptorchidism [50].

The pathogenic variants identified in these genes were reported in NCBI and their causal role has been shown by functional studies [29,30,31,32]. In particular, two of these variants in homozygosis were identified in *TEX11* gene in case 1 (c.2288T > C and c.776C > T), of which one pathogenic and another likely benign. The pathogenic variant c.2288T > C identified in case 1 has been described in the literature in association with azoospermia with functional studies [29].

Three variants of *CCDC39* gene in heterozygosity were identified in the case 2. The c.610-2A> G variant was considered pathogenic since it alters a splicing acceptor site and has already been reported in the literature associated with the subject’s condition [29]. c.2431C > T variant was of uncertain pathogenic significance because it has not been reported in the literature in association with pathological phenotypes and it affects a conserved residue of the protein altering its nature. Finally, the *CCDC39* c.545C > G variation was a benign variant.

Case 3 was found to be carrier of variants in *CHD7* gene. The heterozygous variant c.2750C > in *CHD7* is a missense variant in a gene region coding for a “critical” portion of the protein whose involvement has already been to be pathogenic [30,36], and it is a variant considered “likely pathogenic” in most of the simulation programs selected for interpretive use. Other pathogenic variants were identified in *NR5A1* gene that is notoriously involved in male infertility. In particular, 2 heterozygous variants in *NR5A1* gene were found in two patients suspected of having a defect of primary spermatogenesis due to mutation in single genes: c.1052C > T in the case 4 and c.1063G > A in the case 5. Case 4 carried the c.1052C > T variant that affects a conserved residue of the protein located in a functional domain. This variant was reported in a patient with bilateral anorchia, in a functional study that demonstrates its reduced transcriptional activity. Furthermore, a segregation study showed that it was also present in the healthy relatives, suggesting a possible effect of incomplete penetrance [32]. Instead, the variation c.1063G > A identified in the case 5 affects a conserved protein residue but not altering its nature. It is in a functional domain of the protein and it has already been reported in the literature in a case of disorders of sexual development [31]. Noteworthy, a different variation of the same codon was reported in association with disorders of sexual development with male karyotype (sex reversal) with functional supporting studies that show that the variant alters a domain functional protein [51].

NGS-based genetic screening of a large panel of genes increases the number of novel variants and variants of uncertain pathogenic significance [52]. In this study, 4 splice variants (c.2316 + 1G > A in *CATSPER1*, c.1600-1G > A in *DRC1*, c.2427 + 1G > A in *HFM1*, c.325 + 2T > C in *USP9Y*) and 3 missense variants (c.2500A > T in *AR*, c.5805G > C in *DNAH11* and c.907T > C in *TUBB8*) predicted in silico as probably deleterious were identified (ST1); however more data are needed to demonstrate their pathogenicity. Therefore, we did not report them in the results section. Surely, they warrant further studies in the future. Among variants with an uncertain pathogenic role, 14 with uncertain pathogenic significance were identified in this study. In the three positive cases, the variants with uncertain pathogenic significance c.776C > T in *TEX1* and c.50G > C in *ZBPB*, c.2431C > T in *CCDC3* and c.614C > T in *CDC151*, c.1106C > T in *FLRT3* were, respectively, identified. In other patients examined, c.6173C > T in *DNAH11*, c.1960C > T in *DNAI1*, c.854C > T in *DNAI2*, c.17G > A in *PLK4* variants with uncertain pathogenic significance and c.2159G > A in *GLI2* and c.1331C > T in *LRRC6* variants with conflicting interpretations of pathogenicity were found. Although including variant of unknown significance (VUS) as decisive results of phenotype in routine clinical elective genetic testing is not recommended, it is recognized that clinical information provided with the sample may give significance to the VUS variants. Anyway, functional studies and segregation studies in other family members could shed light on the clinical significance of novel and with uncertain pathogenic significance variants. Other variants identified were very interesting but it is necessary a functional and segregation family study for these variants, for instance a frameshift variant c.3792_3793del (rs778085976) in *CENPF*, a stop lost variant c.417A > G (rs1429366684) in *KISS1* and numerous splice variants (Supplementary Table S1). Because some cases of male infertility are due to unknown genetic variants, the detection of numerous genetic variants by NGS-based genetic screening is important to reduce the percentage of idiopathic infertility.

Genetic analysis is generally performed using many different platforms. The use of a NGS custom panel is advantageous because it allows the identification of variants by a single platform with optimal sensitivity and specificity. However, there are some limitations. For instance, the NGS custom-made panel cannot detect balanced translocations, which are known to cause infertility in 0.9% of patients and complex chromosomal rearrangements in infertile male patients [53,54]. Anyway, the NGS custom-made panel developed in this study had the ability to provide detailed information that help the clinical management of the infertile male.

In conclusion, our study showed the efficacy of an NGS-based approach in the diagnosis of male infertility according to the patient’s clinical features. In fact, for patients with suspect and established diagnosis of male infertility obtained by first/second level diagnostic tests, it is of pivotal importance to use a genetic test in the attempt of explaining the underlying etiology of the disorder and guide the clinical management. Our gene panel solved the 22% (5/22) of cases considered with apparently idiopathic infertility. This is relevant since the prevalence of idiopathic male infertility has been esteemed as high as the ~70% when panels currently available in the clinical practice are used [55]. Hence, the prevalence could be lower if properly tested with advanced gene panels.

In conclusion, for the patients with a negative diagnosis we could not determine the causative mutations and more studies are needed to evaluate whether the VUS we identified can have an effect on the observed phenotype or other genes should be investigated. Finally, it is not possible to establish phenotypic parameters that would suggest a genetic involvement. Therefore, it is useful to perform genetic testing in all idiopathic infertility cases, regardless of the severity.

## 4. Materials and Methods

### 4.1. Patients and Blood Samples

Twenty-two subjects with a clinical diagnosis of male infertility were selected for this study. All enrolled patients underwent pre-test counseling during which they were informed about the significance of molecular screening providing information about their personal and familial history, and informed consent was obtained from each subject. The study was conducted in accordance with the tenets of the Declaration of Helsinki and it was approved by the local Ethics Committee. A blood EDTA (Ethylenediaminetetraacetic acid) sample was collected from each subject. Samples of genomic DNA of all subjects were extracted from peripheral blood using a commercial kit (SAMAG 120 BLOOD DNA Extraction Kit, Como, Italy). DNA was quantified using Quant-iT Picogreen dsDNA Assay Kit (Invitrogen, Eugene, OR) and a Varioskan LUX (Thermo Scientific, Vantaa Finland).

### 4.2. Gene Selection and Panel Design

A NGS panel consisting of 110 diagnostic genes related to male infertility disorders was designed. The genes included in the panel were based on their correlation with male infertility described in OMIM [17], GeneReviews [56] and primary literature. For each subject, only the genes related to the diagnostic suspect were considered because the analysis was for diagnostic purposes. The custom Illumina Nextera panel included genomic targets comprising coding exons and 15 bp flanking regions of each genes. The list of genes associated with male infertility included in the NGS panel broken down by diagnostic suspect is shown in Table 4. The association of diagnostic suspects with their etiology is shown in the Supplementary Tables (ST2, ST3, ST4, ST5, ST6). The cumulative target length of the gene panel was 314,814 bp. For each diagnostic suspect the size target was respectively: 93,474 bp, 59,379 bp, 3003 bp, 12,729 bp and 162,534 bp.

### 4.3. Genetic Analysis and Variant Detection

DNA samples were processed for library preparation targeted capture and sequencing as previous reported [57,58]. In particular, 150 bp paired-end reads sequencing was performed on MiSeq personal sequencer (Illumina, San Diego, CA) according to the manufacturer’s instructions. Fastq (forward-reverse) files were obtained after sequencing. Reads alignment was done by the BWA (0.7.17-r1188) software. Duplicates were removed using the SAMBAMBA (0.6.7) program and GATK (4.0.0.0) were used for re-alignment. We used international databases dbSNP (www.ncbi.nlm.nih.gov/SNP/) and Human Gene Mutation Database professional (HGMD; http://www.biobase international.com/product/hgmd) for all nucleotide changes. In silico evaluation of the pathogenicity of nucleotide changes in exons was performed using PolyPhen-2 (http://genetics.bwh.harvard.edu/pph2/), SIFT (https://sift.bii.a-star.edu.sg/), and Mutation Taster (http://www. mutationtaster.org). Minor allele frequencies (MAF) were checked in the Genome Aggregation Database (gnomAD (http://gnomad.broadinstitute.org/)). Sanger sequencing was performed for confirmation when target region coverage was less than 10 reads. Nucleotide alterations were analyzed and validated by Sanger sequencing. After confirmation, each variant was classified as pathogenic, likely pathogenic, VUS, likely benign, or benign, in according to the American College of Medical Genetics (ACMG) guidelines [59]. Variants were considered causative when at least 2 predictors evaluated the variants as damaging and/or there were literature data that demonstrated the pathogenicity.

## Figures and Tables

**Table 1:** Variants identified in the patients with a positive genetic diagnosis.

Case	Gene (Transcript Isoform)	Variant Position	Variant	Zygosity	SNP ID	Polyphen-2	SIFT	Mutation Taster	MAF (%)	Ref
1	*TEX11* (NM_001003811.1)	chrX:70554698	c.2288T > C; p.Val763Ala	Hem	rs200139216	Benign	Damaging	Polymorphism	0.02%	[28]
2	*CCDC39* (NM_181426.1)	chr3:180616671	c.2431C > T; p.Arg811Cys	Het	rs574993914	Probably damaging	Damaging	Disease-causing	0.007%	/
		chr3:180659582	c.610–2A > G	Het	rs756235547	/	/	Disease-causing	0.01%	[29]
3	*CHD7* (NM_017780.3)	chr8:60821842	c.2750C > T; p.Thr917Met	Het	rs1165711448	Probably damaging	Damaging	Disease-causing	/	[30]
4	*NR5A1* (NM_004959.4)	chr9:124491167	c.1052C > T; p.Ala351Val	Het	rs759071081	Probably damaging	Tolerated	Disease-causing	0.005%	[31]
5	*NR5A1* (NM_004959.4)	chr9:124491156	c.1063G > A; p.Val355Met	Het	rs371701248	Probably damaging	Damaging	Disease-causing	0.01%	[31]

**Table 2:** NGS Coverage performance for positive subjects of the diagnostic test.

	Medium Coverage	Coverage >10X
Case 1	317.7X	98.5%
Case 2	296.5X	98.7%
Case 3	539.2X	98.9%
Case 4	296.5X	98.7%
Case 5	286.7X	91.3%

**Table 3:** Available clinical characteristics of all the analyzed patients. Abbreviations: FSH, follicle-stimulating hormone; * evaluated with ultrasound; ** values before therapy.

	Diagnostic Suspect	Sperm Parameters	FSH (IU/L)	Mean Testicular Volume (mL) *	Additional Data
Case 1	Primary spermatogenic defects	Oligoasthenozoospermia (6.6 mil/ejac)	8.1	14.4	//
Case 2	Primary ciliary dyskinesia	Azoospermia, then oligoasthenoteratozoospermia after scleroembolization	Unavailable	Unavailable	Right varicocele, dextrocardia, bronchiectasia, abnormal connecting piece, and absence of outer dynein arms in the sperm axoneme, abnormal number and position of peripheral microtubules and central pair in the respiratory cilia
Case 3	Central hypogonadism	Severe oligoasthenoteratozoospermia	Unavailable	5.4	Low gonadotropins, treated with testosterone replacement therapy, severe left varicocele
Case 4	Primary spermatogenic defects	Oligoteratozoospermia (21.5 mil/ejac)	4.1	14.5	//
Case 5	Primary spermatogenic defects	Oligoasthenozoospermia (11.5 mil/ejac)	17.5	8	Testicular hypotrophy
Case 6	Primary spermatogenic defects	Severe oligoasthenoteratozoospermia (<5 mil/ejac)	6.11	12.7	The patient was treated with FSH to increase the sperm number, but was unresponsive
Case 7	Primary spermatogenic defects	Unavailable	Unavailable	Unavailable	//
Case 8	Primary spermatogenic defects	Azoospermia	3.24	14.8	The patient was counseled for testicular sperm extraction, with no result
Case 9	Congenital hypopituitarism	Azoospermia **	1.67 **	12.0	The patient was counseled for therapy with gonadotropins. The couple opted for ART (Assisted Reproductive Technology) using sperm of a donor. Hence, testosterone was prescribed
Case 10	Primary spermatogenic defects	Mild oligozoospermia	3.77	11.6	//
Case 11	Primary spermatogenic defects	Azoospermia	30.9	6.6	Sertoli cell only syndrome was found in testicular histology
Case 12	Central hypogonadism	Criptozoospermia **	<0.9 **	5.5	The patient was responsive to treatment with gonadotropins
Case 13	Androgen insensitivity	Unavailable	Unavailable	Unavailable	The patient had abnormally high testosterone serum level. He has normal androgenization
Case 14	Primary spermatogenic defects	Severe oligoasthenoteratozoospermia (<5 mil/ejac)	2.14	8.0	//
Case 15	Primary spermatogenic defects	Moderate oligoasthenozoospermia (<15 mil/ejac)	6.9	11.5	The patient was unresponsive to treatment with FSH
Case 16	Primary spermatogenic defects	Unavailable	Unavailable	Unavailable	//
Case 17	Primary spermatogenic defects	Unavailable	Unavailable	Unavailable	//
Case 18	Primary spermatogenic defects	Unavailable	Unavailable	Unavailable	//
Case 19	Primary spermatogenic defects	Unavailable	Unavailable	Unavailable	//
Case 20	Primary spermatogenic defects	Unavailable	Unavailable	Unavailable	//
Case 21	Primary spermatogenic defects	Unavailable	Unavailable	Unavailable	//
Case 22	Primary spermatogenic defects	Unavailable	Unavailable	Unavailable	//

**Table 4:** Genes associated with male infertility included in the custom NGS panel.

Male Condition	Location	*Genes*	OMIM #	REFSEQ	Gene Name	% Target Bases with Coverage ≥ 10X
Defects of primary spermatogenesis	19q13.43	*AURKC*	603495	NM_001015878	aurora kinase C	100.0%
	11q13.1	*CATSPER1*	606389	NM_053054	cation channel sperm associated 1	100.0%
	3q13.2	*CFAP44*	617559	NM_018338	cilia and flagella-associated protein 44	100.0%
	12q14.2	*DPY19L2*	613893	NM_173812	dpy-19 like 2	100.0%
	17q21.2	*KLHL10*	608778	NM_152467	kelch like family member 10	100.0%
	10q26.11	*NANOS1*	608226	NM_199461	nanos C2HC-type zinc finger 1	39.45%
	22q13.1	*PICK1*	605926	NM_012407	protein interacting with PRKCA 1	99.75%
	4q28.1	*PLK4*	605031	NM_014264	polo like kinase 4	100.0%
	16p13.3	*SEPTIN12*	611562	NM_144605	septin 12	100.0%
	9q34.3	*SOHLH1*	610224	NM_001012415	spermatogenesis and oogenesis-specific basic helix-loop-helix 1	100.0%
	20q11.21	*SUN5*	613942	NM_080675	Sad1 and UNC84 domain-containing 5	85.74%
	12q23.2	*SYCP3*	604759	NM_001177948	synaptonemal complex protein 3	99.69%
	Xp11	*TEX11*	300311	NM_001003811	testis-expressed 11	96.43%
	Yq11.221	*USP9Y*	400005	NM_004654	ubiquitin specific peptidase 9 Y-linked	99.8%
	7p12.2	*ZPBP*	608498	NM_007009	zona pellucida binding protein	98.08%
	1p22.1	*BRDT*	602144	NM_001726	bromodomain testis-associated	100.0%
	10q25.1	*CFAP43*	617558	NM_025145	cilia and flagella-associated protein 43	98.45%
	3p21.1	*DNAH1*	603332	NM_015512	dynein axonemal heavy chain 1	100.0%
	6q22.31	*HSF2*	140581	NM_004506	heat shock transcription factor 2	100.0%
	16p13.3	*MEIOB*	617670	NM_152764	meiosis specific with OB-fold	100.0%
	9q33.3	*NR5A1*	184757	NM_004959	nuclear receptor subfamily 5 group A member 1	100.0%
	12p12.3	*PLCZ1*	608075	NM_033123	phospholipase C zeta 1	100.0%
	Xq24	*RHOXF2*	300447	NM_032498	Rhox homeobox family member 2	95.41%
	6p21.31	*SLC26A8*	608480	NM_052961	solute carrier family 26 member 8	100.0%
	3q26.31	*SPATA16*	609856	NM_031955	spermatogenesis associated 16	100.0%
	10q26.3	*SYCE1*	611486	NM_130784	synaptonemal complex central element protein 1	100.0%
	18q11.2	*TAF4B*	601689	NM_005640	TATA-box binding protein-associated factor 4b	98.17%
	8p12	*TEX15*	605795	NM_001350162	testis-expressed 15, meiosis, and synapsis-associated	100.0%
	17p13.2	*ZMYND15*	614312	NM_001136046	zinc finger MYND-type containing 15	92.29%
Hypogonadotropic hypogonadism	Xp22.31	*ANOS1*	300836	NM_000216	anosmin 1	95.4%
	2q31.2	*CCDC141*	616031	NM_173648	coiled-coil domain-containing 141	99.89%
	12q21.33	*DUSP6*	602748	NM_001946	dual specificity phosphatase 6	100.0%
	8p21.3	*FGF17*	603725	NM_003867	fibroblast growth factor 17	100.0%
	8p11.23	*FGFR1*	136350	NM_023110	fibroblast growth factor receptor 1	100.0%
	11p14.1	*FSHB*	136530	NM_000510	follicle-stimulating hormone subunit beta	100.0%
	4q13.2	*GNRHR*	138850	NM_000406	gonadotropin-releasing hormone receptor	100.0%
	3p14.3	*IL17RD*	606807	NM_017563	interleukin 17 receptor D	100.0%
	19p13.3	*KISS1R*	604161	NM_032551	KISS1 receptor	84.84%
	9q34.3	*NSMF*	608137	NM_015537	NMDA receptor synaptonuclear signaling and neuronal migration factor	95.03%
	3p13	*PROK2*	607002	NM_021935	prokineticin 2	97.67%
	7q21.11	*SEMA3A*	603961	NM_006080	semaphorin 3A	100.0%
	9q34.3	*SOHLH1*	610224	NM_001012415	spermatogenesis and oogenesis-specific basic helix-loop-helix 1	100.0%
	5q31.3	*SPRY4*	607984	NM_030964	sprouty RTK-signaling antagonist 4	98.25%
	5q31.3	*SRA1*	603819	NM_001035235	steroid receptor RNA activator 1	100.0%
	4q24	*TACR3*	162332	NM_001059	tachykinin receptor 3	100.0%
	19q13.2	*AXL*	109135	NM_021913	AXL receptor tyrosine kinase	100.0%
	8q12.2	*CHD7*	608892	NM_017780	chromodomain helicase DNA binding protein 7	99.54%
	7q31.32	*FEZF1*	613301	NM_001024613	FEZ family zinc finger 1	96.46%
	10q24.32	*FGF8*	600483	NM_033163	fibroblast growth factor 8	93.16%
	20p12.1	*FLRT3*	604808	NM_198391	fibronectin leucine rich transmembrane protein 3	100.0%
	8p21.2	*GNRH1*	152760	NM_001083111	gonadotropin-releasing hormone 1	100.0%
	2q14.3	*HS6ST1*	604846	NM_004807	heparan sulfate 6-O-sulfotransferase 1	96.3%
	1q32.1	*KISS1*	603286	NM_002256	KiSS-1 metastasis suppressor	100.0%
	19q13.33	*LHB*	152780	NM_000894	luteinizing hormone subunit beta	100.0%
	20p12.3	*PROKR2*	607123	NM_144773	prokineticin receptor 2	100.0%
	7q21.11	*SEMA3E*	608166	NM_012431	semaphorin 3E	100.0%
	22q13.1	*SOX10*	602229	NM_006941	SRY-box transcription factor 10	100.0%
	12q13.3	*TAC3*	162330	NM_013251	tachykinin precursor 3	100.0%
	10q26.12	*WDR11*	606417	NM_018117	WD repeat domain 11	100.0%
Androgen insensitivity	Xq12	*AR*	313700	NM_000044	androgen receptor	100.0%
Congenital hypopituitarism	2q14.2	*GLI2*	165230	NM_005270	GLI family zinc finger 2	91.92%
	9q34.3	*LHX3*	600577	NM_014564	LIM homeobox 3	81.51%
	14q22.3	*OTX2*	600037	NM_172337	orthodenticle homeobox 2	100.0%
	5q35.3	*PROP1*	601538	NM_006261	PROP paired-like homeobox 1	95.99%
	3p14.3	*HESX1*	601802	NM_003865	HESX homeobox 1	100.0%
	1q25.2	*LHX4*	602146	NM_033343	LIM homeobox 4	100.0%
	3p11.2	*POU1F1*	173110	NM_000306	POU class 1 homeobox 1	100.0%
	Xq27.1	*SOX3*	313430	NM_005634	SRY-box transcription factor 3	70.12%
Primary ciliary dyskinesia	10p12.1	*ARMC4*	615408	NM_001290020	armadillo repeat-containing 4	99.65%
	17q21.31	*CCDC103*	614677	NM_213607	coiled-coil domain-containing 103	100.0%
	19p13.2	*CCDC151*	615956	NM_145045	coiled-coil domain-containing 151	99.13%
	17q25.3	*CCDC40*	613799	NM_017950	coiled-coil domain-containing 40	98.52%
	5q11.2	*CCNO*	607752	NM_021147	cyclin O	100.0%
	21q22.11	*CFAP298*	615494	NM_021254	cilia and flagella-associated protein 298	100.0%
	14q21.3	*DNAAF2*	612517	NM_018139	dynein axonemal assembly factor 2	95.63%
	19q13.42	*DNAAF3*	614566	NM_178837	dynein axonemal assembly factor 3	89.24%
	7p22.3	*DNAAF5*	614864	NM_017802	dynein axonemal assembly factor 5	78.93%
	7p15.3	*DNAH11*	603339	NM_001277115	dynein axonemal heavy chain 11	99.94%
	6p21.2	*DNAH8*	603337	NM_001206927	dynein axonemal heavy chain 8	99.93%
	17q25.1	*DNAI2*	605483	NM_023036	dynein axonemal intermediate chain 2	100.0%
	14q24.3	*DNAL1*	610062	NM_031427	dynein axonemal light chain 1	100.0%
	16q24.3	*GAS8*	605178	NM_001481	growth arrest specific 8	98.09%
	8q24.22	*LRRC6*	614930	NM_012472	leucine rich repeat-containing 6	100.0%
	7p14.1	*NME8*	607421	NM_016616	NME/NM23 family member 8	100.0%
	21q22.3	*RSPH1*	609314	NM_080860	radial spoke head component 1	100.0%
	6q22.1	*RSPH4A*	612647	NM_001010892	radial spoke head component 4A	100.0%
	8q22.2	*SPAG1*	603395	NM_172218	sperm associated antigen 1	94.7%
	17q21.2	*TTC25*	617095	NM_031421	tetratricopeptide repeat domain 25	90.46%
	3p14.2	*CFAP20DC*	300572	NM_198463	CFAP20 domain-containing	100.0%
	19q13.33	*CCDC114*	615038	NM_144577	coiled-coil domain-containing 114	100.0%
	3q26.33	*CCDC39*	613798	NM_181426	coiled-coil domain-containing 39	100.0%
	12q13.12	*CCDC65*	611088	NM_033124	coiled-coil domain-containing 65	100.0%
	1q41	*CENPF*	600236	NM_016343	centromere protein F	100.0%
	16q24.1	*DNAAF1*	613190	NM_178452	dynein axonemal assembly factor 1	100.0%
	15q21.3	*DNAAF4*	608706	NM_130810	dynein axonemal assembly factor 4	100.0%
	3p21.1	*DNAH1*	603332	NM_015512	dynein axonemal heavy chain 1	100.0%
	5p15.2	*DNAH5*	603335	NM_001369	dynein axonemal heavy chain 5	99.82%
	9p13.3	*DNAI1*	604366	NM_012144	dynein axonemal intermediate chain 1	100.0%
	11q13.4	*DNAJB13*	610263	NM_153614	DnaJ heat shock protein family (Hsp40) member B13	100.0%
	2p23.3	*DRC1*	615288	NM_145038	dynein regulatory complex subunit 1	93.24%
	16q22.2	*HYDIN*	610812	NM_001270974	HYDIN axonemal central pair apparatus protein	98.92%
	5q11.2	*MCIDAS*	614086	NM_001190787	multiciliate differentiation and DNA synthesis associated cell cycle protein	100.0%
	Xq22.3	*DNAAF6*	300933	NM_001169154	dynein axonemal assembly factor 6	100.0%
	6q25.3	*RSPH3*	615876	NM_031924	radial spoke head 3	100.0%
	6p21.1	*RSPH9*	612648	NM_152732	radial spoke head component 9	100.0%
	2q35	*STK36*	607652	NM_015690	serine/threonine kinase 36	100.0%
	3p21.31	*ZMYND10*	607070	NM_015896	zinc finger MYND-type-containing 10	100.0%

## Supplementary Materials

The following are available online at https://www.mdpi.com/2075-1729/10/10/242/s1, Table S1: Genetic variants identified in the analysed population by NGS. ^1^ All identified variants are indicated both by cDNA base sequence (third column) and by protein sequence (fourth column) according to the HGVS (Human Genome Variation Society) nomenclature guidelines.^2^ Information reported in NCBI (National Centre for Biotechnology Information) database, Table S2: List of defects of primary spermatogenesis due to mutation in single genes and their genetic etiology, Table S3: List of hypogonadotropic hypogonadism and their genetic etiology, Table S4: List of primary ciliary dyskinesia and their genetic etiology, Table S5: List of androgen insensitivity and their genetic etiology, Table S6: List of congenital hypopituitarism and their genetic etiology.
