# Supplementary material for: Clinical Evaluation of a Custom Gene Panel as a Tool for Precision Male Infertility Diagnosis by Next-Generation Sequencing
Source: Life (Basel). 2020 Oct 15;10(10):242. doi: 10.3390/life10100242 (PMC7602585; doi:10.3390/life10100242)
Supplement: Supplementary file 1 [file life-10-00242-s001.pdf]

life

MDPI

Supplementary Materials

Table S1. Genetic variants identified in the analysed population by NGS. [1]All identified variants are indicated both by cDNA base sequence (third column) and by protein sequence (fourth column) according to the HGVS (Human Genome Variation Society) nomenclature guidelines.[2]Information reported in NCBI (National Centre for Biotechnology Information) database.

Gene	Variant Position	HGVS[1] Cdna	HGVS[1] Protein	Reference ID According To NCBI	Consequence	Clinic Relevance[2]
AR	chrX:67722877	NM_000044.4:c.2500A>T	NP_000035.2:p.Asn834Tyr		Missense variant	-
BRDT	chr1:91981022	NM_001242805.2:c.1594C>T	NP_001229734.2:p.Arg532Ter	rs969395495	Stop gained	-
C3orf67	chr3:58863617	NM_001351530.1:c.1369G>A	NP_001338459.1:p.Val457Met	rs34631714	Missense variant	-
CATSPER1	chr11:66017059	NM_053054.3:c.2316+1G>A			Splice donor variant	-
CCDC141	chr2:178905348	NM_001316745.1:c.1246A>G	NP_001303674.1:p.Ser416Gly	rs748639287	Missense variant	-
CCDC39	chr3:180616671	NM_181426.1:c.2431C>T	NP_852091.1:p.Arg811Cys	rs574993914	Missense variant	-
CCDC39	chr3:180659582	NM_181426.1:c.610-2A>G	-	rs756235547	Splice acceptor variant	Pathogenic
CCDC39	chr3:180659741	NM_181426.2:c.545C>G	NP_852091.1:p.Thr182Ser	rs112738198	Missense variant	Benign
CCDC40	chr17:80084742	NM_017950.3:c.1990-1G>C	-	rs746905855	Splice acceptor variant	-
CENPF	chr1:214642128-214642129	NM_016343.3:c.3792_3793del	NP_057427.3:p.Cys1264Ter	rs778085976	frameshift variant	-
CHD7	chr8:60821842	NM_017780.3:c.2750C>T	NP_060250.2:p.Thr917Met	rs1165711448	missense variant	-
DNAH1	chr3:52392896	NM_015512.4:c.10345G>A	NP_056327.4:p.Val3449Met	rs757371106	missense variant	-
DNAH1	chr3:52372236	NM_015512.4:c.6676A>G	NP_056327.4:p.Ile2226Val	rs112505934	missense variant	-
DNAH11	chr7:21698206	NM_001277115.1:c.6173C>T	NP_001264044.1:p.Ser2058Phe	rs765090393	missense variant	Uncertain significance
DNAH11	chr7:21687408	NM_001277115.1:c.5805G>C	NP_001264044.1:p.Leu1935Phe		missense variant	-
DNAH5	chr5:13714412	NM_001369.2:c.13118C>A	NP_001360.1:p.Pro4373His	rs1459001748	missense variant	-
DNAH8	chr6:38886870	NM_001206927.1:c.8339T>C	NP_001193856.1:p.Ile2780Thr	rs142328376	missense variant	Benign
DNAI1	chr9:34517414	NM_001281428.1:c.1960C>T	NP_001268357.1:p.Arg654Cys	rs140820295	missense variant	Uncertain significance
DNAI2	chr17:74299847	NM_001172810.2:c.854C>T	NP_001166281.1:p.Thr285Met	rs141079076	missense variant	Uncertain significance

DRC1	chr2:26450591	NM_145038.4:c.1600-1G>A	-		Splice acceptor variant	-
FGF17	chr8:22046249	NM_003867.3:c.208G>A	NP_003858.1:p.Gly70Arg	rs368521586	missense variant	-
FLRT3	chr20:14326401	NM_198391.2:c.1106C>T	NP_938205.1:p.Ala369Val	rs771836971	missense variant	-
GLI2	chr2:120986480	NM_005270.4:c.2159G>A	NP_005261.2:p.Arg720His	rs149091975	missense variant	Likely benign or Uncertain significance
HFM1	chr1:91343457	NM_001017975.4:c.2308G>A	NP_001017975.4:p.Asp770Asn	rs143399622	missense variant	Benign
HFM1	chr1:91324674	NM_001017975.4:c.2427+1G>A	-		Splice donor variant	-
HYDIN	chr16:70872081	NM_001270974.2:c.11047C>T	NP_001257903.1:p.Arg3683Trp	rs200260585	missense variant	-
KISS1	chr1:204190484	NM_002256.3:c.417A>G	NP_002247.3:p.Ter139TrpextTer41	rs1429366684	stop lost	-
LRRC6	chr8:132572316	NM_001321961.1:c.1331C>T	NP_001308890.1:p.Pro444Leu	rs139131485	missense variant	Likely benign or Uncertain significance
NR5A1	chr9:124491156	NM_004959.4:c.1063G>A	NP_004950.2:p.Val355Met	rs371701248	missense variant	-
NR5A1	chr9:124491167	NM_004959.4:c.1052C>T	NP_004950.2:p.Ala351Val	rs759071081	missense variant	-
NSMF	chr9:137457758	NM_001130969.1:c.277G>A	NP_001124441.1:p.Ala93Thr	rs753078916	missense variant	-
NSMF	chr9:137452768	NM_001130969.1:c.1099C>T	NP_001124441.1:p.Arg367Trp	rs772028925	missense variant	-
PATL2	chr15:44669839	NM_001145112.1:c.814G>A	NP_001138584.1:p.Val272Met	rs140098598	missense variant	-
PLK4	chr4:127881151	NM_014264.4:c.17G>A	NP_055079.3:p.Gly6Glu	rs149003893	missense variant	Uncertain significance
SEPT12	chr16:4777907	NM_001154458.2:c.829C>T	NP_001147930.1:p.Arg277Cys	rs764712886	missense variant	-
TEX11	chrX:70554698	NM_001003811.1:c.2288T>C	NP_001003811.1:p.Val763Ala	rs200139216	missense variant	-
TEX11	chrX:70744181	NM_001003811.1:c.776C>T	NP_001003811.1:p.Thr259Ile	rs762957753	missense variant	-
TUBB8	chr10:47485	NM_177987.2:c.907T>C	NP_817124.1:p.Cys303Arg		missense variant	-
USP9Y	chrY:12722189	NM_004654.3:c.325+2T>C	-		Splice donor variant	-
ZMYND10	chr3:50341826	NM_015896.3:c.1105C>T	NP_056980.2:p.Arg369Trp	rs142613783	missense variant	Benign

Table S2. List of defects of primary spermatogenesis due to mutation in single genes and their genetic etiology.

Location	Gene	Inheritance	OMIM # Gene	Phenotype	Spermatogenic Defect	OMIM# Phenotype /HGMD Reference
19q13.43	*AURKC (STK13)*	AR	603495	Spermatogenic failure 5	Large-headed, multiflagellar, polyploid spermatozoa (Macrozoospermia)	243060
1p22.1	*BRDT*	AR	602144	Spermatogenic failure 21	Acephalic spermatozoa syndrome	617644
11q13.1	*CATSPER1*	AR	606389	Spermatogenic failure 7	Asthenozoospermia	612997
10q25.1	*CFAP43*	AR	617558	Spermatogenic failure 19	MMAF	617592
3q13.2	*CFAP44*	AR	617559	Spermatogenic failure 20	MMAF	617593
3p21.1	*DNAH1*	AR	603332	Spermatogenic failure 18	MMAF	617576
12q14.2	*DPY19L2*	AR	613893	Spermatogenic failure 9	Globozoospermia	613958
6q22.31	*HSF2*	AD	140581	-	Idiopathic azoospermia	Mou et al (2013)
17q21.2	*KLHL10*	AD	608778	Spermatogenic failure 11	OZS; Teratozoospermia; AZS	615081
16p13.3	*MEIOB*	AR	617670	Spermatogenic failure 22	Azoospermia, non-obstructive	617706
10q26.11	*NANOS1*	AD	608226	Spermatogenic failure 12	Azoospermia/ Oligozoospermia/ Oligoasthenoteratozoospermia	615413
9q33.3	*NR5A1*	AR	184757	Spermatogenic failure 8	Azoospermia/ Oligozoospermia	613957
22q13.1	*PICK1*		605926	-	Globozoospermia	Liu et al (2010)
12p12.3	*PLCZ1*	AR	608075	Spermatogenic failure 17	Male infertility due to oocyte activation failure	617214
4q28.1	*PLK4*	AD	605031	-	Azoospermia Sertoli cell-only	Miyamoto et al (2016)
6p21.31	*SEPT12*	AD	611562	Spermatogenic failure 10	Asthenozoospermia, oligoasthenozoospermia e Teratozoospermia	614822
9q34.3	*SLC26A8*	AD	608480	Spermatogenic failure 3	Asthenozoospermia	617187
3q26.31	*SOHLH1*	-	610224	-	Azoospermia, non-obstructive	Choi et al (2010)
20q11.21	*SPATA16*	AR	609856	Spermatogenic failure 6	Globozoospermia	102530
10q26.3	*SUN5*	AR	613942	Spermatogenic failure 16	Acephalic spermatozoa syndrome	617187
12q23.2	*SYCE1*	AR	611486	Spermatogenic failure 15	Azoospermia, non-obstructive	616950
18q11.2	*SYCP3*	AD	604759	Spermatogenic failure 4	Azoospermia/ Oligozoospermia	270960
Xp11	*TAF4B*	AR	601689	Spermatogenic failure 13	Azoospermia/ Oligozoospermia	615841
8p12	*TEX11*	XLR	300311	Spermatogenic failure, X-linked, 2	Azoospermia	309120
Yq11.221	*TEX15*	AR	605795	-	Azoospermia/ Oligozoospermia	Okutman (2015); Colombo (2017); Wang (2017)
17p13.2	*USP9Y*	YL	400005	Spermatogenic failure, Y-linked, 2	Azoospermia, non-obstructive	415000

7p12.2	*ZMYND15*	AR	614312	Spermatogenic failure 14	Azoospermia/ Oligozoospermia	615842
19q13.43	*ZPBP*	-	608498	-	Teratozoospermia	Yatsenko et al (2012)

OZS: oligozoospermia; AZS: asthenozoospermia; MMAF: Multiple Morphological Abnormalities of the Flagella; AR = autosomal recessive; AD = autosomal dominant; XLR= X-linked, recessive; YL: Y-linked.

Table S3. List of hypogonadotropic hypogonadism and their genetic etiology.

Location	Phenotype	OMIM# Phenotype / HGMD Reference	Inheritance	Gene	OMIM# Gene
Xp22.31	Hypogonadotropic hypogonadism, 1 with or without anosmia (Kallmann syndrome 1, KS)	308700	XLR	*ANOS1 (KAL1)*	300836
19q13.2	Hypogonadotropic hypogonadism	Salian-Mehta (2014)	-	*AXL*	109135
2q31.2	Kallmann syndrome	Hutchins [2016]	-	*CCDC141*	616031
8q12.2	Hypogonadotropic hypogonadism, 5 with or without anosmia	612370	AD	*CHD7*	608892
12q21.33	Hypogonadotropic hypogonadism, 19 with or without anosmia	615269	AD	*DUSP6*	602748
7q31.32	Hypogonadotropic hypogonadism, with or without anosmia	616030	AR	*FEZF1*	613301
8p21.3	Hypogonadotropic hypogonadism, 20 with or without anosmia	615270	AD	*FGF17*	603725
10q24.32	Hypogonadotropic hypogonadism, 6 with or without anosmia	612702	AD	*FGF8*	600483
8p11.23	Hypogonadotropic hypogonadism, 2 with or without anosmia	147950	AD	*FGFR1*	136350
20p12.1	Hypogonadotropic hypogonadism, 21 with anosmia	615271	AD	*FLRT3*	604808
11p14.1	Hypogonadotropic hypogonadism, 24 without anosmia	229070	AR	*FSHB*	136530
8p21.2	Hypogonadotropic hypogonadism, 12 with or without anosmia	614841	AR	*GNRH1*	152760
4q13.2	Hypogonadotropic hypogonadism, 7 without anosmia	146110	AR	*GNRHR*	138850
2q14.3	Hypogonadotropic hypogonadism, 15 with or without anosmia	614880	AD	*HS6ST1*	604846

3p14.3	Hypogonadotropic hypogonadism, 18 with or without anosmia	615267	AD	*IL17RD*	606807
1q32.1	Hypogonadotropic hypogonadism, 13 with or without anosmia	614842	AR	*KISS1*	603286
19p13.3	Hypogonadotropic hypogonadism, 8 with or without anosmia	614837	AR	*KISS1R*	604161
19q13.33	Hypogonadotropic hypogonadism, 23 with or without anosmia	228300	AR	*LHB*	152780
9q34.3	Hypogonadotropic hypogonadism, 9 with or without anosmia	614838	AD	*NSMF*	608137
3p13	Hypogonadotropic hypogonadism, 4 with or without anosmia	610628	AD	*PROK2*	607002
20p12.3	Hypogonadotropic hypogonadism, 3 with or without anosmia	244200	AD	*PROKR2*	607123
7q21.11	Hypogonadotropic hypogonadism, 16 with or without anosmia	614897	AD	*SEMA3A*	603961
7q21.11	Kallmann syndrome	Cariboni (2015)	-	*SEMA3E*	608166
9q34.3	Hypergonadotropic hypogonadism, nonsyndromic	Bayram (2015)	-	*SOHLH1*	610224
22q13.1	Kallmann syndrome, with or without deafness	Pingault (2013); Marcos (2014)	-	*SOX10*	602229
5q31.3	Hypogonadotropic hypogonadism, 17 with or without anosmia	615266	AD	*SPRY4*	607984
5q31.3	Hypogonadotropic hypogonadism	Kotan (2016)	-	*SRA1*	603819
12q13.3	Hypogonadotropic hypogonadism, 10 with or without anosmia	614839	AR	*TAC3*	162330
4q24	Hypogonadotropic hypogonadism, 11 with or without anosmia	614840	AR	*TACR3*	162332
10q26.12	Hypogonadotropic hypogonadism, 14 with or without anosmia	614858	AD	*WDR11*	606417

HGMD = Human Gene Mutation Database (https://portal.biobase-international.com/hgmd/pro/start.php?); AR = Autosomal recessive; AD = autosomal dominant; XLR= X-linked, recessive.

Table S4. List of primary ciliary dyskinesia and their genetic etiology.

Location	Phenotype	OMIM# Phenotype / HGMD Reference / Reference	Inheritance	Gene	OMIM# Gene
10p12.1	Ciliary dyskinesia, primary, 23	615451	AR	*ARMC4*	615408
21q22.11	Ciliary dyskinesia, primary, 26	615500	AR	*CFAP298*	615494
3p14.2	Ciliary dyskinesia, primary	Abouelhoda (2016)	-	*CFAP20DC (C3orf67)*	-
17q21.31	Ciliary dyskinesia, primary, 17	614679	AR	*CCDC103*	614677
19q13.33	Ciliary dyskinesia, primary, 20	615067	AR	*CCDC114*	615038
19p13.2	Ciliary dyskinesia, primary, 30	616037	AR	*CCDC151*	615956
3q26.33	Ciliary dyskinesia, primary, 14	613807	-	*CCDC39*	613798
17q25.3	Ciliary dyskinesia, primary, 15	613808	-	*CCDC40*	613799
12q13.12	Ciliary dyskinesia, primary, 27	615504	AR	*CCDC65*	611088
5q11.2	Ciliary dyskinesia, primary, 29	615872	AR	*CCNO*	607752
1q41	Ciliopathy & microcephaly	Waters (2015)	-	*CENPF*	600236
16q23.3-q24.1	Ciliary dyskinesia, primary, 13	613193	AR	*DNAAF1*	613190
14q21.3	Ciliary dyskinesia, primary, 10	612518	-	*DNAAF2*	612517
19q13.42	Ciliary dyskinesia, primary, 2	606763	AR	*DNAAF3*	614566
15q21.3	Ciliary dyskinesia, primary, 25	615482	AR	*DNAAF4*	608706
Unknown	Ciliary dyskinesia, primary, 18	614874	AR	*DNAAF5*	614864
3p21.1	Ciliary dyskinesia, primary, 37	617577	AR	*DNAH1*	603332
7p15.3	Ciliary dyskinesia, primary, 7, with or without situs inversus	611884	AR	*DNAH11*	603339
5p15.2	Ciliary dyskinesia, primary, 3, with or without situs inversus	608644	-	*DNAH5*	603335
6p21.2	Stromme syndrome; Ciliary dyskinesia, primary	243605	-	*DNAH8*	603337
9p13.3	Ciliary dyskinesia, primary, 1, with or without situs inversus	244400	AR	*DNAI1*	604366
17q25.1	Ciliary dyskinesia, primary, 9, with or without situs inversus	612444	-	*DNAI2*	605483
11q13.4	Ciliary dyskinesia, primary, 34	617091	AR	*DNAJB13*	610263
14q24.3	Ciliary dyskinesia, primary, 16	614017	AR	*DNAL1*	610062
2p23.3	Ciliary dyskinesia, primary, 21	615294	AR	*DRC1*	615288
16q24.3	Ciliary dyskinesia, primary, 33	616726	AR	*GAS8*	605178
16q22.2	Ciliary dyskinesia, primary, 5	608647	AR	*HYDIN*	610812
8q24.22	Ciliary dyskinesia, primary, 19	614935	AR	*LRRC6*	614930
5q11.2	Reduced generation of multiple motile cilia; Primary ciliary dyskinesia	Boon (2014); Maddirevula (2018)	-	*MCIDAS*	614086
7p14.1	Ciliary dyskinesia, primary, 6	610852	AR	*NME8*	607421
Xq22.3	Ciliary dyskinesia, primary, 36, X-linked	300991	XLR	*DNAAF6 (PIH1D3)*	300933
21q22.3	Ciliary dyskinesia, primary, 24	615481	AR	*RSPH1*	609314
6q25.3	Ciliary dyskinesia, primary, 32	616481	AR	*RSPH3*	615876
6q22.1	Ciliary dyskinesia, primary, 11	612649	-	*RSPH4A*	612647
6p21.1	Ciliary dyskinesia, primary, 12	612650	-	*RSPH9*	612648
8q22.2	Ciliary dyskinesia, primary, 28	615505	AR	*SPAG1*	603395
2q35	Primary ciliary dyskinesia	Edelbusch (2017)	-	*STK36*	607652
17q21.2	Ciliary dyskinesia, primary, 35	617092	AR	*TTC25*	617095
3p21.31	Ciliary dyskinesia, primary, 22	615444	AR	*ZMYND10*	607070

HGMD = Human Gene Mutation Database (https://portal.biobase-international.com/hgmd/pro/start.php?); AR = Autosomal recessive; AD = autosomal dominant; XLR= X-linked, recessive.

Table S5. List of androgen insensitivity and their genetic etiology.

Location	Phenotype	OMIM# Phenotype / HGMD Reference	Inheritance	Gene	OMIM# Gene
Xq12	Androgen insensitivity	300068	XLR	*AR*	313700

XLR= X-linked, recessive.

Table S6. List of congenital hypopituitarism and their genetic etiology.

Location	Phenotype	OMIM# Phenotype / HGMD Reference	Inheritance	Gene	OMIM# Gene
5q35.3	CPHD, 2; Panhypopituitarism	262600	AR	*PROP1*	601538
Xq27.1	Panhypopituitarism, X-linked	312000	XL	*SOX3*	313430
3p11.2	CPHD, 1	613038	AR, AD	*POU1F1*	173110
3p14.3	CPHD, 5	182230	AR, AD	*HESX1*	601802
1q25.2	CPHD, 4	262700	AD	*LHX4*	602146
9q34.3	CPHD, 3	221750	AR	*LHX3*	600577
14q22.3	CPHD, 6	613986	AD	*OTX2*	600037
2q14.2	CPHD	Babu (2019)	-	*GLI2*	165230

HGMD = Human Gene Mutation Database (https://portal.biobase-international.com/hgmd/pro/start.php?); CPHD= Pituitary hormone deficiency, combined; AR = autosomal recessive; AD = autosomal dominant.
